# An in vitro cell model for exploring inflammatory and amyloidogenic events in alkaptonuria

**DOI:** 10.1002/jcp.31449

**Published:** 2024-10-01

**Authors:** Pierfrancesco Mastroeni, Michela Geminiani, Tommaso Olmastroni, Luisa Frusciante, Alfonso Trezza, Anna Visibelli, Annalisa Santucci

**Affiliations:** ^1^ Dipartimento di Biotecnologie, Chimica e Farmacia Università di Siena Siena Italy; ^2^ MetabERN, Dipartimento di Biotecnologie, Chimica e Farmacia Università di Siena Siena Italy

**Keywords:** alkaptonuria, homogentisic acid, inflammation, metabolic disease, ochronotic pigment, oxidative stress

## Abstract

Alkaptonuria (AKU) is a progressive systemic inherited metabolic disorder primarily affecting the osteoarticular system, characterized by the degeneration of cartilage induced by ochronosis, ultimately leading to early osteoarthritis (OA). However, investigating AKU pathology in human chondrocytes, which is crucial for understanding the disease, encounters challenges due to limited availability and donor variability. To overcome this obstacle, an in vitro model has been established using homogentisic acid (HGA) to simulate AKU conditions. This model employed immortalized C20/A4 human chondrocytes and serves as a dependable platform for studying AKU pathogenesis. Significantly, the model demonstrates the accumulation of ochronotic pigment in HGA‐treated cells, consistent with findings from previous studies. Furthermore, investigations into inflammatory processes during HGA exposure revealed notable oxidative stress, as indicated by elevated levels of reactive oxygen species and lipid peroxidation. Additionally, the model demonstrated HGA‐induced inflammatory responses, evidenced by increased production of nitric oxide, overexpression of inducible nitric oxide synthase, and cyclooxygenase‐2. These findings underscore the model's utility in studying inflammation associated with AKU. Moreover, analysis of serum amyloid A and serum amyloid P proteins revealed a potential interaction, corroborating evidence of amyloid fibril formation. This hypothesis was further supported by Congo red staining, which showed fibril formation exclusively in HGA‐treated cells. Overall, the C20/A4 cell model provided valuable insights into AKU pathogenesis, emphasizing its potential for facilitating drug development and therapeutic interventions.

## 1 INTRODUCTION

Alkaptonuria (AKU) is an exceptionally rare autosomal recessive genetic disorder (Bernardini et al., 2024) caused by a deficiency in the enzyme homogentisate 1,2−dioxygenase (HGD). This deficiency leads to the accumulation of homogentisic acid (HGA) due to impaired catabolism of tyrosine and phenylalanine. The oxidized derivative of HGA, benzoquinone acetic acid, forms polymer deposits that accumulate in connective tissues resulting in a pigmentation condition known as “ochronosis.” This condition causes widespread deposition of ochronotic pigment in various organs, exacerbating organ dysfunction (Phornphutkul et al. 2002) and increasing tissue oxidative stress through the generation of reactive oxygen species (ROS).

Ochronotic arthropathy is the most common clinical manifestation of AKU, characterized by cartilage degeneration, chronic inflammation, and pain (Helliwell et al., 2008). Despite the recent approval of nitisinone therapy (Ranganath et al., 2020), treatment remains focused on symptom management, often requiring total joint replacement in advanced stages. Studies have shown that HGA induces significant protein oxidation in serum and chondrocytes (Spreafico et al., 2013a), leading to oxidative stress and chondroptosis, a specific form of apoptosis observed in AKU cartilage (Millucci, Giorgetti, et al., 2015). AKU has also been associated with secondary amyloidosis (Millucci et al., 2012; Millucci, Braconi, et al., 2015; Millucci, Ghezzi, Bernardini, et al., 2014; Millucci, Ghezzi, Braconi, et al., 2014; Millucci, Ghezzi, Paccagnini, et al., 2014), particularly AA amyloidosis, where serum amyloid A (SAA) fibrils deposit in tissues (Momohara et al., 2008), potentially leading to organ failure. Amyloid deposits have been identified in alkaptonuric tissue, likely due to localized HGD expression within the osteoarticular system (Bernardini et al., 2012; Laschi et al., 2012). Moreover, pigment deposition in AKU tissues co‐localizes with SAA‐amyloid (Braconi et al., 2011, 2012; Braconi, Laschi, Amato, et al., 2010; Braconi, Laschi, Taylor, et al., 2010; Tinti et al., 2010, 2011), implicating a role in secondary amyloidosis. Further research into AKU mechanisms has revealed structural and functional alterations in chondrocytes, including abnormal cytoskeletal protein expression (Geminiani et al., 2017), oxidative stress‐induced dysfunction (Braconi et al., 2015, 2016; Braconi, Laschi, Amato, et al., 2010), reduced primary cilia length, and disrupted Hedgehog signaling (Gambassi et al., 2017; Thorpe et al., 2017). Autophagy, essential for cellular homeostasis, is progressively impaired by HGA‐induced oxidative stress, worsening chondrocyte death (Galderisi et al., 2022). Challenges in developing AKU therapies include the ultra‐rare nature of the disease, the lack of severe symptoms in early childhood, and decreasing research investment. Additionally, limited access to biological samples complicates research efforts. To overcome these challenges, in vitro models using human chondrocytes cultured with HGA have been developed (Tinti et al., 2010), aiding in understanding ochronotic pigment deposition mechanisms and facilitating therapeutic strategies. The C20/A4 cell line has emerged as a valuable model for studying AKU in vitro (Braconi, Laschi, Taylor, et al., 2010), providing rapid findings compared to primary cells and eliminating the need for biopsy sample extraction.

The aim of this study was to develop a novel cell model utilizing the C20/A4 human chondrocyte cell line to investigate inflammatory responses in AKU. Our results not only confirmed the reliability of this model for studying AKU, but also demonstrated its efficacy in assessing inflammatory processes. Specifically, we observed an increased production of nitric oxide (NO), accompanied by the overexpression of inducible nitric oxide synthase (iNOS), cyclooxygenase‐2 (COX‐2) and 4‐hydroxynonenal (4‐HNE) proteins in C20/A4 cells treated with HGA. Additionally, the investigation of SAA and serum amyloid P (SAP) proteins revealed a potential relationship in HGA‐treated C20/A4 cells, further supported by Congo red staining, indicating amyloid fibril production.

## 2 MATERIALS AND METHODS

### 2.1 Cell cultures and treatments

The immortalized human chondrocyte cell line C20/A4 (Sigma‐Aldrich, SCC041) was cultured at 37℃ in a humidified atmosphere with 5% CO_2_ using Dulbecco's modified Eagle's medium supplemented with 1% penicillin/streptomycin and 10% fetal bovine serum. A 10 mM stock solution of HGA (Sigma Aldrich) was prepared by dissolving HGA in deionized water. This stock solution was then diluted in the cell culture medium to achieve final concentrations of 0.066 and 0.1 mM, reflecting levels observed in AKU patients (Tinti et al., 2011). These concentrations were selected for treatments lasting 1–2 weeks for subsequent experiments.

### 2.2 Fontana‐Masson stain

C20/A4 cells were seeded in a 6‐well plate at a density of 2 × 10^5^ cells per well and allowed to grow until reaching 70%–80% confluence. The cells were then treated with HGA at concentrations of 0.066 and 0.1 mM for 14 days. After treatment, cells were washed, fixed in 75% ethanol, stained with ammoniacal silver solution, fixed again with 2% sodium thiosulfate and finally dehydrated with ethanol. The stained samples were observed under a bright‐field microscope. For pigment quantification, ImageJ software was utilized, employing the Measure function. The obtained values were normalized against the area to ensure accurate quantification.

### 2.3 Detection of amyloid deposits

To investigate the distribution of presumptive amyloidosis, C20/A4 cells were seeded into 6‐well plates at a density of 2 × 10^5^ cells per well and cultured until reaching sub‐confluence (80%–85%). Subsequently, the cells were treated with HGA at varying concentrations (0.066 mM, 0.1 mM) for 14 days. Cells were then stained with 1% Congo Red and dehydrated using ethanol, followed by examination under a bright‐field microscope. The samples were then further analyzed under polarized light using a Zeiss Axio Lab A1 microscope to detect any birefringence indicative of amyloid fibrils.

### 2.4 Quantification of ochronotic pigment deposition by autofluorescence

For fluorescence‐based pigment detection, C20/A4 cells were seeded in a 24‐well plate at a density of 2 × 10^4^ cells per well for a 1‐week treatment, and 1 × 10^4^ cells per well for a 2‐week treatment. The cells were allowed to grow until reaching 70%–80% confluence and were then treated with HGA at concentrations of 0.066 mM and 0.1 mM HGA for 7 and 14 days. Following the treatment period, the cells were fixed in 4% formaldehyde. Glass coverslips were then mounted using Fluoroshield mounting medium (Abcam). Images were captured using a Zeiss AxioLabA1 fluorescence microscope equipped with a FITC filter. Fluorescence intensity was quantified using the Measure function in ImageJ software, and the obtained values were normalized against the area to ensure accurate quantification.

### 2.5 Quantification of intracellular ROS formation

ROS production in C20/A4 cells was assessed in 96‐well plates using 2′,7′‐dichlorodihydrofluorescein diacetate (DCFH_2_‐DA, Sigma‐Aldrich), which is intracellularly deacetylated and oxidized to highly fluorescent 2′,7′‐dichlorofluorescein (DCF) (Ng & Ooi, 2021). The cells were treated with HGA at a concentration of 0.066 mM for 24 h. DCFH_2_‐DA (5 µM), dissolved in Hank's Balanced Salt Solution (HBSS), was then added to the cells and incubated for 10 min at 37℃. Fluorescence was measured using an EnVision system (PerkinElmer) with excitation at 485 nm and emission at 535 nm. To visualize DCF fluorescence, samples were observed under a fluorescence microscope with a FITC filter (Zeiss AxioLabA1), and images were acquired to confirm the results. The number of cells in each well was then determined using a Crystal Violet assay (Feoktistova et al., 2016). Results were normalized to the relative cell count for each well and expressed as relative ROS production compared to the untreated group.

### 2.6 Determination of NO production

To evaluate NO production in the supernatant of C20/A4 cells, a Griess assay was performed. Cells were seeded in 6‐well plates at a density of 2 × 10^6^ cells per well and cultured until they reached sub‐confluence (80%–85%). The cells were then treated with HGA at different concentrations (0.066 mM, 0.1 mM) for 14 days. Subsequently, 100 µL of conditioned medium from each well was mixed with an equal volume of Griess reagent, consisting of 1% sulfanilamide and 0.1% N‐(1‐naphthyl) ethylenediamine dihydrochloride in 5% phosphoric acid. The absorbance was measured at 540 nm using an EnVision system (PerkinElmer). Nitrite concentration was determined by comparing the absorbance values to a sodium nitrite standard curve.

### 2.7 Western blot analysis

Cells at 75%–80% confluence were subjected to the aforementioned treatment protocol. Following treatment, the cells were washed, and cellular lysates were prepared using RIPA buffer supplemented with phosphate and protease inhibitors. The lysates were then disrupted by sonication in an ice bath. Subsequently, 20 μg of protein from each sample were separated by 8% sodium dodecyl sulfate‐polyacrylamide gel electrophoresis and transferred onto a nitrocellulose membrane. The primary antibodies utilized were as follows: anti‐iNOS (rabbit polyclonal IgG, Sigma‐Aldrich), anti‐GAPDH HRP‐conjugated, anti‐SAA (Mouse monoclonal IgG, ThermoFisher Scientific), anti‐4‐HNE (Mouse monoclonal IgG, Acris), and anti‐COX‐2 (rabbit polyclonal IgG, Sigma‐Aldrich). For secondary antibody detection, anti‐rabbit HRP‐conjugated antibody and anti‐mouse HRP‐conjugated antibody (both from Sigma‐Aldrich) were used. The immunoreactive bands were visualized using enhanced chemiluminescence with Luminata Crescendo (Merck Millipore), and images were captured using a LAS4000 machine (GE Healthcare). The optical densities of the immunoreactive bands were analyzed using ImageQuantTL software V 7.0 (GE Healthcare). Protein levels were normalized against GAPDH (Glyceraldehyde 3‐phosphate dehydrogenase), which served as a protein‐loading control.

### 2.8 Immunofluorescence study

C20/A4 cells were seeded onto glass coverslips at a density of 2 × 10^4^ cells for 1 week and 1 × 10^4^ cells for 2 weeks. After treatment, cells were fixed with methanol, permeabilized with pure acetone, and then blocked. The cells were then incubated overnight at 4℃ with the following primary antibodies: anti‐SAA mouse monoclonal antibody (1:100, Sigma‐Aldrich), anti‐SAP rabbit monoclonal antibody (1:30, Sigma‐Aldrich), and anti‐4‐HNE mouse monoclonal antibody (1:50, Invitrogen). Afterwards, the cells were incubated with secondary antibodies: Alexa Fluor™ 546 goat anti‐mouse Ig (1:100, Thermo Fisher Scientific) and Alexa Fluor™ 488 goat anti‐rabbit Ig (1:100, Thermo Fisher Scientific). The cells were then mounted with Fluoroshield mounting medium containing DAPI (Abcam). Images were captured using a fluorescence microscope (Zeiss AxioLabA1).

### 2.9 Statistical analysis

The experiments were conducted in triplicate. Statistical analyses were carried out using GraphPad Prism 9.0 software (GraphPad Software). The data are expressed as mean ± standard deviation (SD) and were compared using either the unpaired *t*‐test or one‐way analysis of variance (ANOVA) followed by an appropriate post hoc test. A *p*‐value of 0.05 or less was considered statistically significant.

## 3 RESULTS

### 3.1 A substantial accumulation of ochronotic pigment was observed in the C20/A4 model following HGA treatment

A characteristic feature of AKU is the formation of intracellular ochronotic pigment. To track pigment deposition over time, C20/A4 cells treated with HGA were stained using the melanin‐specific Fontana Masson staining technique. Concentrations of 0.066 mM and 0.1 mM HGA were selected to define the C20/A4 AKU model (Galderisi et al., 2022). The ochronotic pigment, identifiable by black‐colored spots, was observed in both intracellular and extracellular compartments after 2 weeks of treatment with both concentrations.

**Figure 1:**
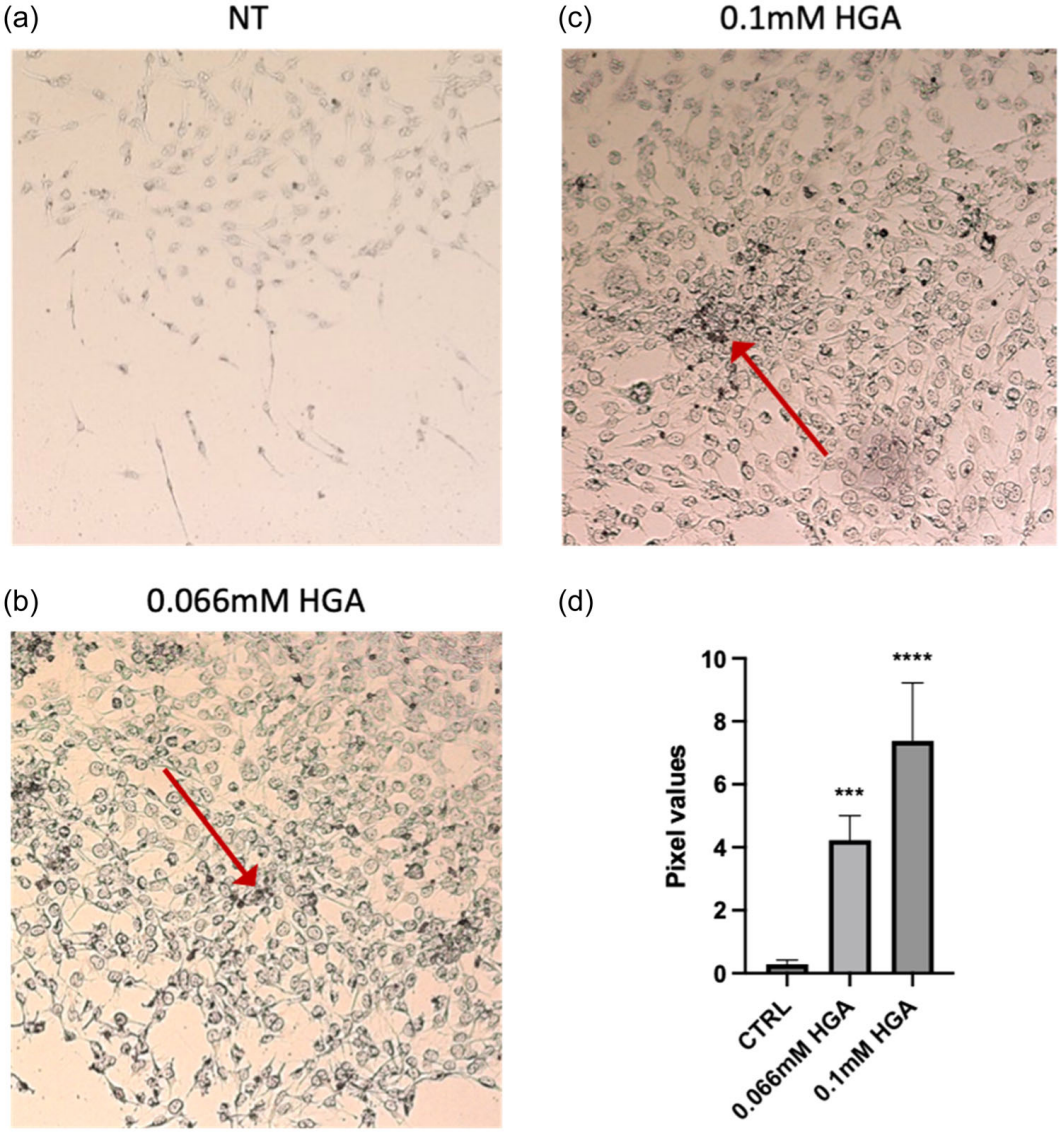
Accumulation of ochronotic pigment (arrow) in C20/A4 cells treated with (b) 0.066 mM and (c) 0.1 mM HGA for 2 weeks. (a) C20/A4 cells without any treatment. Fontana Masson staining revealed black pigmentation indicative of pigment deposition. Cells are shown at 10× magnification. (d) Quantification of black pigmentation intensity was performed by ImageJ software and was normalized to cellular area. Statistically significant differences are denoted by ***p = 0.0003 and ****p < 0.0001 (vs. CTRL). All experiments were performed in triplicate. Data are reported as fold change. *p*‐values were calculated, by ordinary one‐way ANOVA with Dunnett's post hoc test.

The results, presented as mean pixel values of pigment deposition relative to the control, revealed a concentration‐dependent increase in pigment deposition (Figure 1).

**Figure 2:**
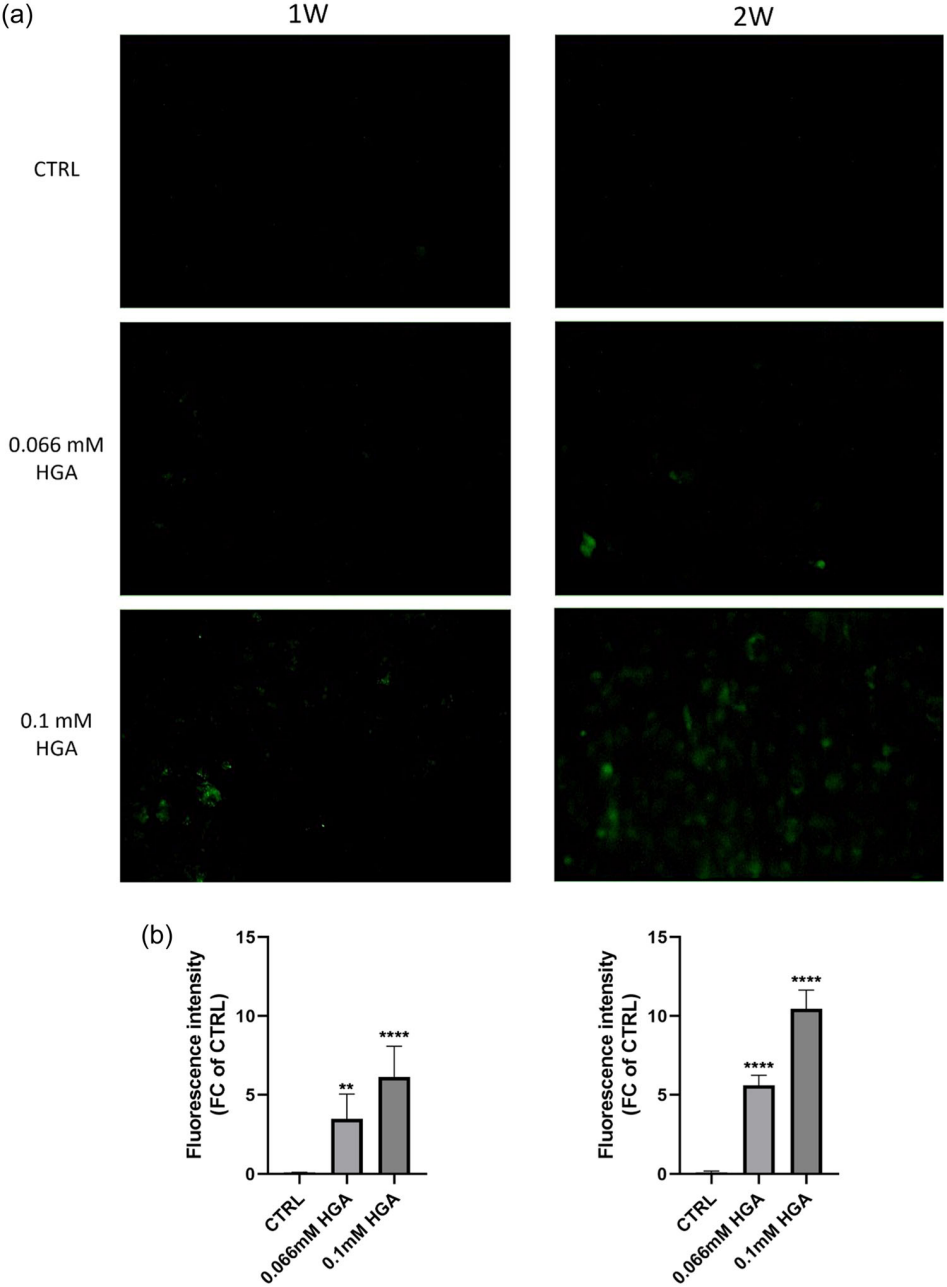
(a) Time‐varying ochronotic pigment formation in C20/A4 cells treated with 0.066 and 0.1 mM HGA for one and 2 weeks detected by formaldehyde fixation. Cells are shown at 40× magnification. (b) Graphical representation of fluorescence intensity. Statistically significant differences are denoted by **p = 0.002 and ****p < 0.0001 (vs. CTRL). All experiments were performed in triplicate. Data are reported as fold change. *p*‐values were calculated, by ordinary one‐way ANOVA with Dunnett's post hoc test.

Furthermore, formaldehyde‐induced fluorescence was utilized to detect melanin‐like ochronotic pigment. Autofluorescence provides a valuable method for studying various pigments, including melanin and melanin‐like substances. Melanin, a complex biopolymer responsible for pigmentation in the skin, hair, and eyes, exhibits intrinsic autofluorescence. Notably, this property is enhanced when melanin is exposed to formaldehyde. The reaction between melanin and formaldehyde results in increased fluorescence, facilitating easier identification and analysis in histological examinations (Hara et al., 2016). Similarly, melanin‐like pigments, such as ochronotic pigment, also display autofluorescent properties (Galderisi et al., 2022). Utilizing autofluorescence to study this pigment provides an effective approach for visualizing and evaluating its deposition in cells and tissues, thereby aiding in the diagnosis and understanding of disease progression. Ochronotic pigment autofluorescence, induced by fixation with formaldehyde, became evident in C20/A4 cells containing ochronotic pigment after 1 week of treatment with 0.066 and 0.1 mM HGA and it exhibited significantly higher levels after 2 weeks of treatment at both concentrations (Figure 2).

The quantitative analysis of fluorescence intensity revealed an increase in pigmentation that was dependent on both the duration of treatment and the concentrations of HGA. This increase was observed in both intracellular and extracellular compartments after 1 week and, particularly, after 2 weeks of treatment.

### 3.2 HGA induced aggregation of amyloidogenic proteins

**Figure 3:**
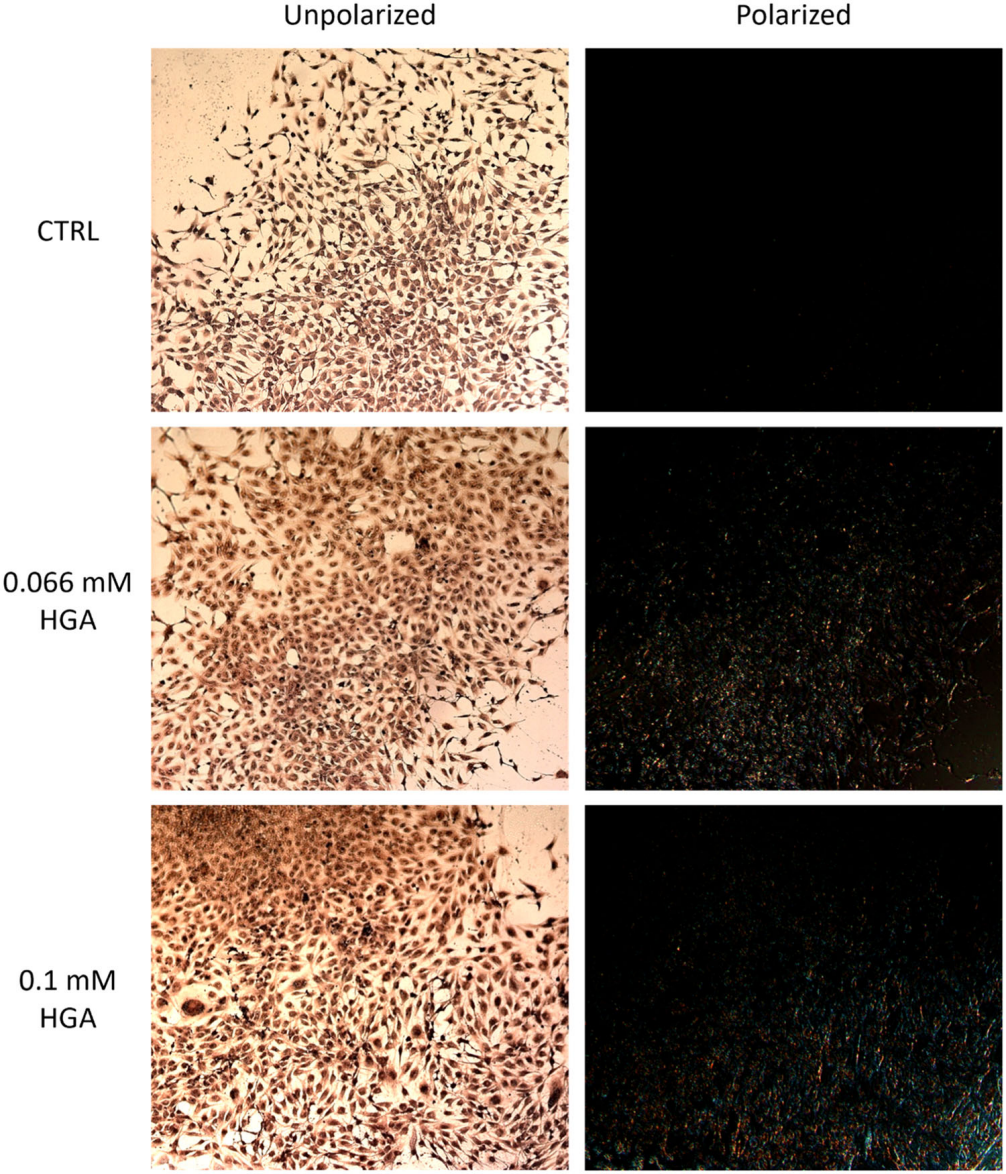
Assessment of amyloid presence in C20/A4 cells treated with 0.066 and 0.1 mM HGA for 2 weeks by Congo Red staining. Cells are shown at 10× magnification.

Knowledge about SAA and SAP content in AKU has been established, allowing the disease to be classified as a secondary amyloidosis (Millucci et al., 2012). Congo red is an efficient staining method for visualizing amyloid components due to its special affinity for amyloid fibrils structures. When bound to amyloid, Congo red exhibits birefringence under polarized light, displaying a characteristic green‐yellow color. This optical property, combined with its high binding affinity, makes Congo Red a reliable and widely used tool in the diagnosis and study of amyloidosis. Under polarized light, the staining in C20/A4 cells treated for 2 weeks with 0.066 and 0.1 mM HGA revealed green‐yellow birefringence, whereas the control cells did not display such characteristics (Figure 3). The presence of amyloid deposits in HGA‐treated cells appeared to be linked to a simulated disease advancement, as untreated cells showed no evidence of deposition.

### 3.3 Detection of SAA and SAP in HGA‐treated C20/A4 cells

**Figure 4:**
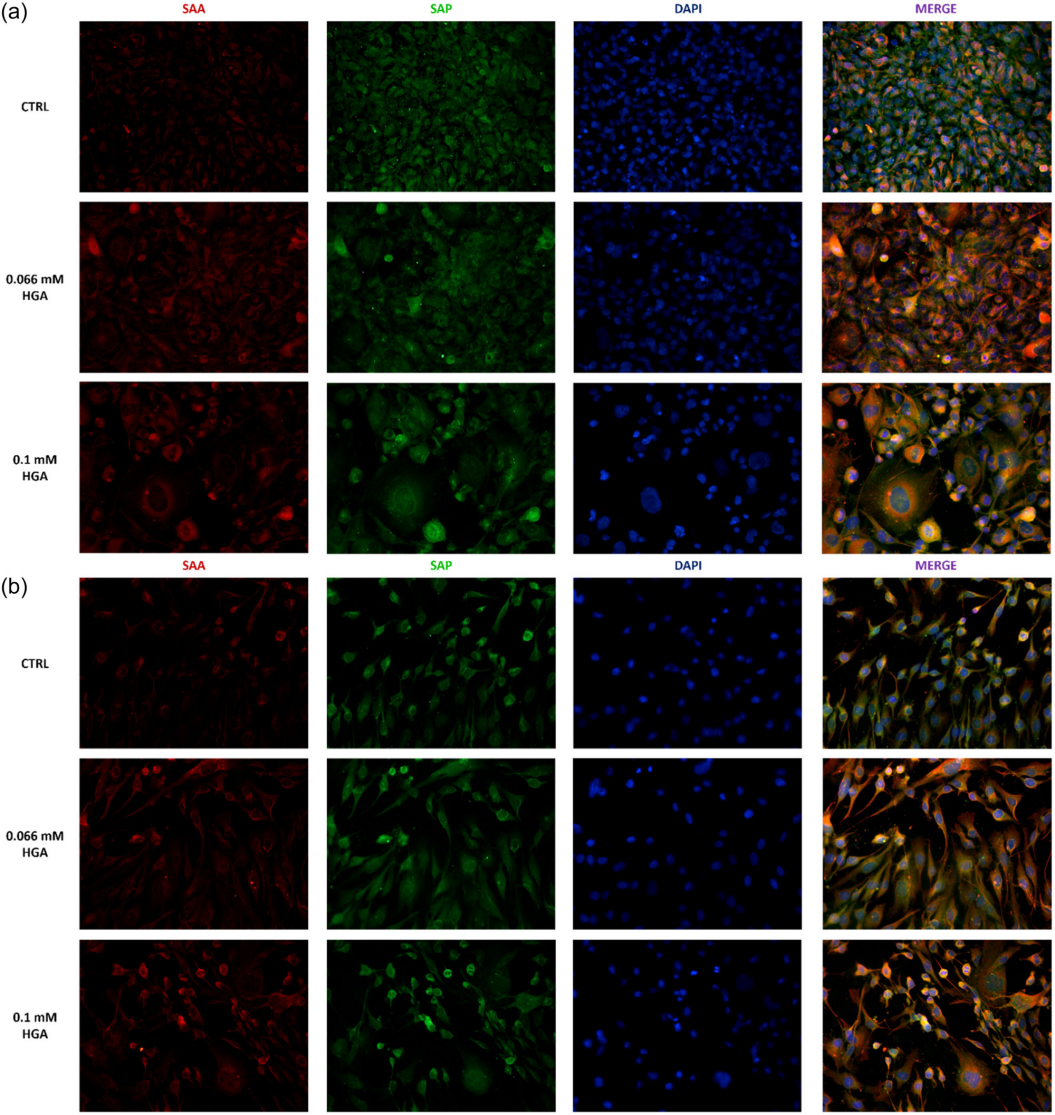
Double labeling of SAA (red) and SAP markers (green) in control and 0.066 mM, 0.1 mM HGA treated cells. Cells are shown at 40× magnification.

To investigate the presence of SAA and SAP proteins, immunofluorescence staining was conducted on HGA‐treated and control C20/A4 cells for 1 and 2 weeks. Both SAA and SAP were detectable in control and treated cells (Figure 4), exhibiting clear and intense red (SAA) and green (SAP) staining. In untreated cells, the two proteins seemed to be equally distributed. However, in cells treated with 0.066 mM HGA, and particularly at 0.1 mM, both SAA and SAP were primarily localized in the perinuclear zone. After 1 week (Figure 4a) and 2 weeks (Figure 4b) of HGA treatment, C20/A4 cells also began to lose their structure, showing alterations in nuclear shape, with indistinct boundaries and morphology, as well as chromatin condensation. Additionally, observations indicated a tendency for the two signals to overlap in HGA‐treated C20/A4 cells, a trend that was less evident in the control samples after 1or 2 weeks.

### 3.4 HGA induced pronounced oxidative stress

Since HGA is known to cause oxidation in chondrocytes, leading to oxidative stress and chondroptosis (Millucci et al., 2015), we aimed to investigate its effect during both the acute and chronic inflammatory phases. To do this, we treated C20/A4 human chondrocytes with 0.066 mM HGA, a concentration consistent with levels observed in alkaptonuric patients (Ranganath et al., 2016). This dosage effectively replicated disease‐related events without compromising cell viability (Galderisi et al., 2022).

**Figure 5:**
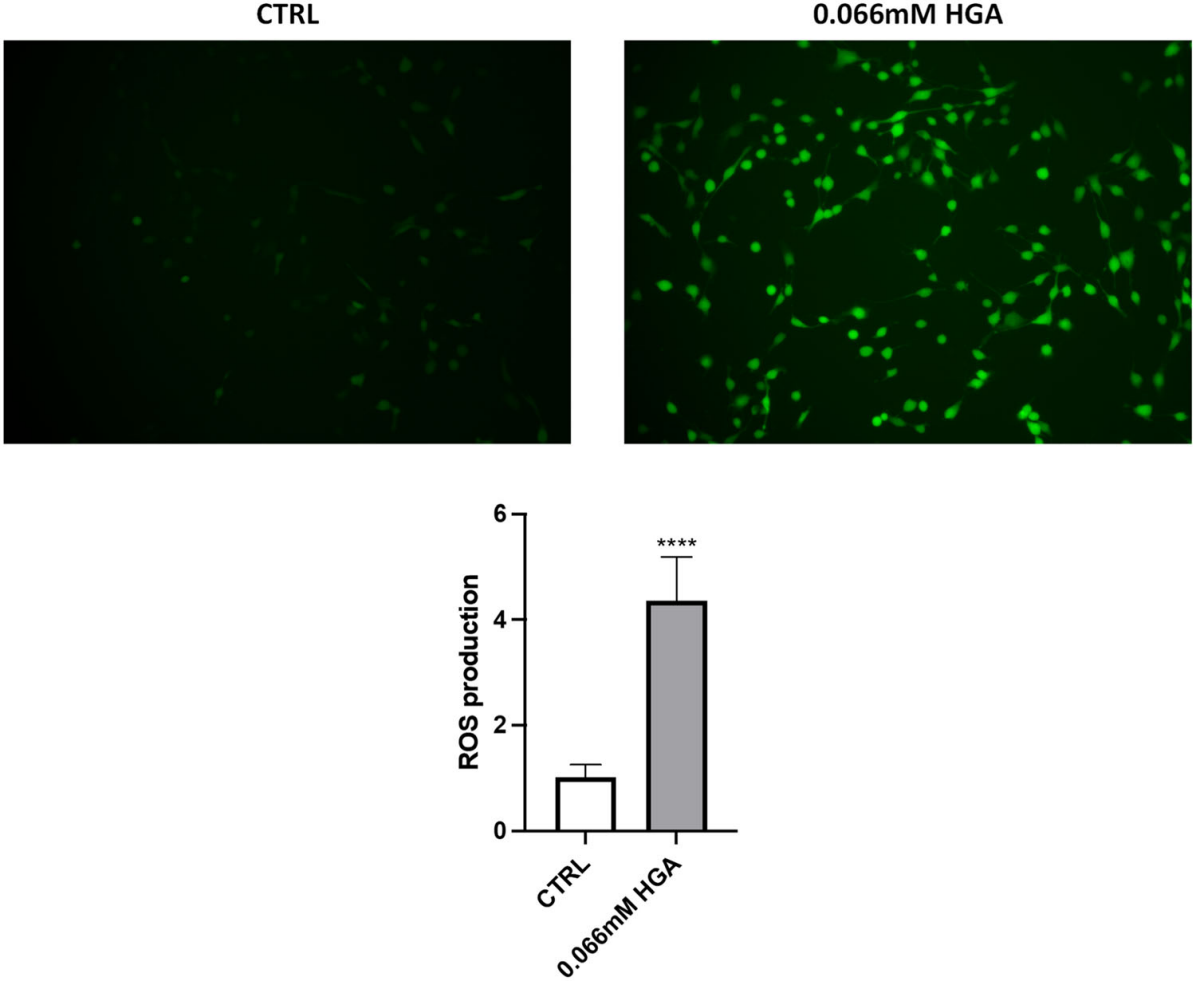
Exploration of ROS generation by labeling with the H2DCFDA probe (green fluorescence) of C20/A4 control and 0.066 mM HGA treated cells. Data are presented as bar graphs for ROS levels measured from fluorescence intensity normalized to cell count with Crystal Violet assay. All experiments were performed in triplicate. Data are reported as fold change. Unpaired *t*‐test was used to assess statistically significant differences, ***p < 0.001. Cells are shown at 20× magnification.

**Figure 6:**
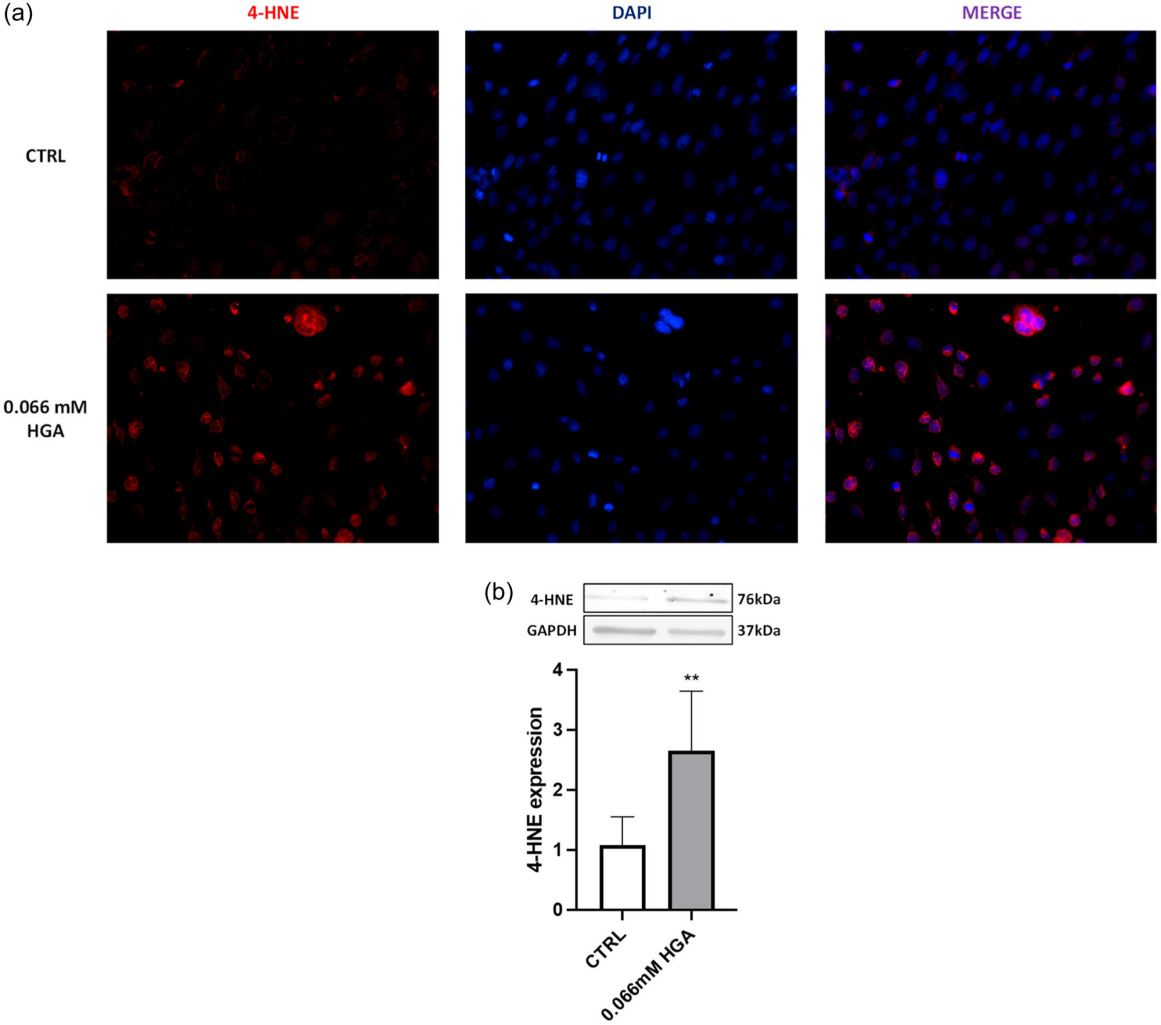
(a) Examination of 4‐HNE expression in control and HGA‐treated C20/A4 chondrocytes after 2 weeks of treatment with 0.066 mM HGA by immunofluorescence staining. Nuclei were counterstained with DAPI. (b) Detection of 4‐HNE in C20/A4 cells through Western blot analysis. Quantification of relative band intensities was determined from three independent experiments. Data are reported as fold change. Unpaired *t*‐test was used to assess statistically significant differences, **p = 0.0026. Cells are shown at 40× magnification.

ROS serve as signaling molecules that play a critical role in the development of inflammatory disorders and are typically produced as part of the early response to stress conditions (Forrester et al., 2018). In chondrocytes, ROS production is also involved in regular processes, such as the regulation of the homeostasis of extracellular matrix (Henrotin et al., 2003). Therefore, we investigated the impact of treating C20/A4 cells with HGA on intracellular ROS production. The DCF fluorescence exhibited a notable increase in HGA‐treated cells compared to the control, indicating the induction of oxidative stress even after 24 h (Figure 5). Long‐term oxidative stress usually results in chronic disorders characterized by damage to DNA and lipidic structures, resulting in structural changes and loss of stability (Galderisi et al., 2022). To evaluate the presence of major products of lipid peroxidation (LPO), 4‐HNE expression was investigated. The fluorescence signal detected in HGA‐treated C20/A4 cells (Figure 6a) revealed a significant presence of LPO, contrasting with control cells.

The findings are consistent with those observed in Western blot analysis, wherein 4‐HNE expression revealed LPO‐induced damage to the cellular lipid structures (Figure 6b).

### 3.5 HGA‐induced inflammatory effects

**Figure 7:**
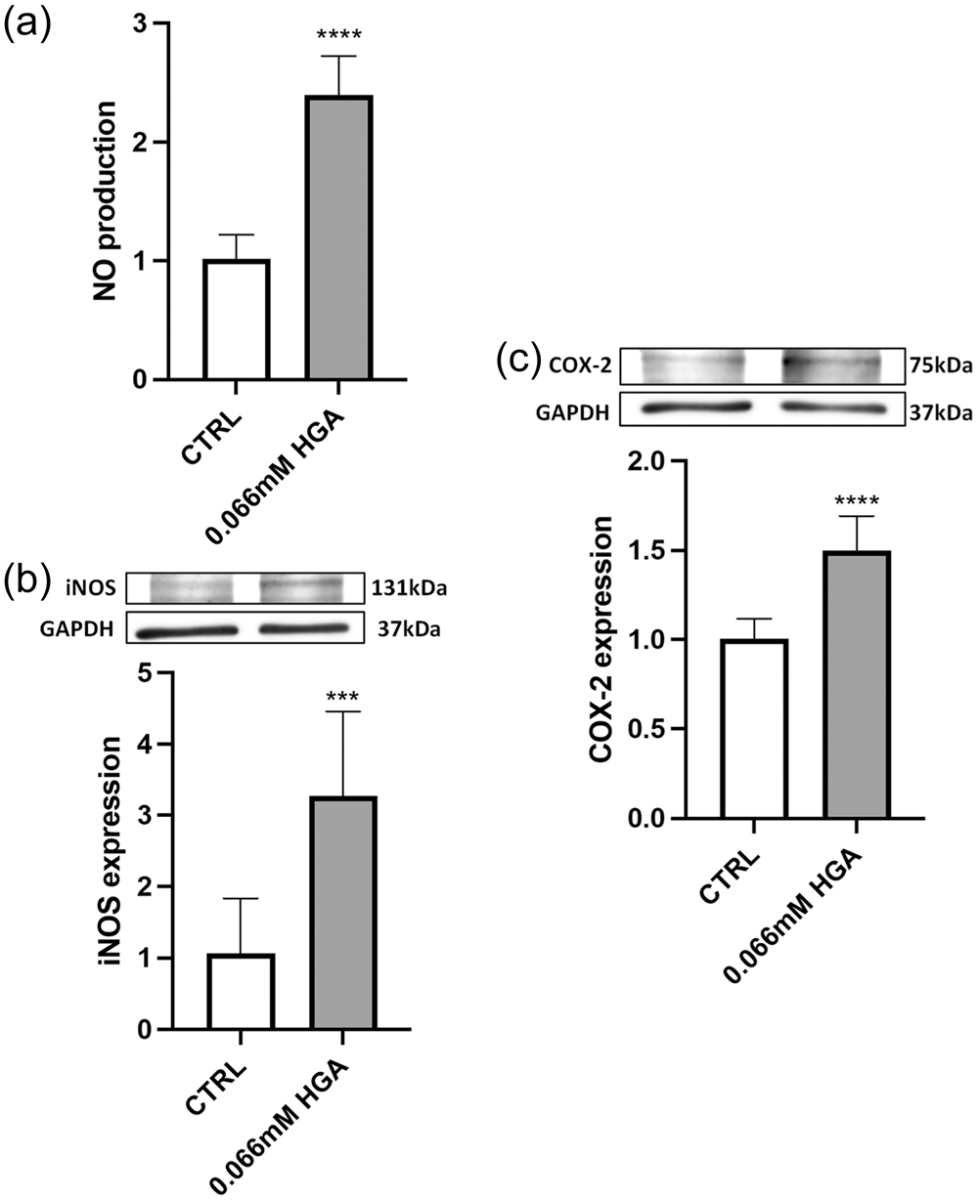
Evaluation of NO production and iNOS and COX‐2 expression in 0.066 mM HGA treated C20/A4 chondrocytes over a 2‐week treatment period. (a) Supernatants from cell cultures were analyzed to assess NO levels. iNOS (b) and COX‐2 (c) expression levels were ascertained via Western blot analysis. Data are reported as fold change. All experiments were performed in triplicate. Unpaired *t*‐test was used to assess statistically significant differences, ***p = 0.0006 and ****p < 0.0001.

According to recent studies, AKU has been linked to a chronic inflammatory state (Braconi et al., 2016). The presence of HGA and its oxidized byproducts in connective tissues, along with a state of oxidative stress and amyloid deposition, promotes an inflammatory response, which contributes to joint and cartilage deterioration, as well as other systemic implications (Braconi, Laschi, Amato, et al., 2010). To investigate inflammatory events in AKU, C20/A4 cells were treated with HGA at a concentration of 0.066 mM for 2 weeks and the levels of key pro‐inflammatory mediators were quantified. The production of NO, detected using the Griess assay, was significantly higher in HGA‐treated cells (Figure 7a) compared to the control.

The protein expression of iNOS and COX‐2 was evaluated via Western blot analysis. Quantitative analysis of immunoreactive bands revealed an upregulation in both iNOS and COX‐2 expression in comparison to the control (Figure 7b,c).

## 4 DISCUSSION

AKU is a complex degenerative disorder that significantly affects multiple organ systems, particularly the osteoarticular system, with cartilage being notably more susceptible to damage compared to bone (Fisher & Davis, 2004). Ochronosis, an essential characteristic of AKU, entails a cascade of effects including weakening of cartilage, increased stiffness, risen susceptibility to fractures, chronic inflammation, and eventual onset of OA (Gallagher et al., 2015).

Chondrocytes, the only cells in cartilage, play a pivotal role in orchestrating the synthesis of extracellular matrix components such as collagen and proteoglycans. These components are essential for maintaining the structural integrity and biomechanical resilience of cartilage, which is important for withstanding mechanical stresses. Nevertheless, conducting research on human chondrocytes faces numerous obstacles, primarily due to the difficulties in obtaining a sufficient number of primary cells from a single source. These challenges are further exacerbated by inherent variations among donors, which can be attributed to factors such as age and concurrent medical conditions. Given the scarcity of AKU cases, obtaining biological specimens for research purposes is not only rare but also extremely difficult to get in controlled experimental settings. This limits the ability to compare and apply research findings to a wider population. Generally, the investigation of events related to AKU has been conducted by analyzing cells directly obtained from biopsies of AKU patients or by using cellular models that replicated the disease condition. The use of an immortalized cell line of human chondrocytes as a cellular model for AKU has provided a novel opportunity for investigating this rare disease (Braconi, Laschi, Taylor, et al., 2010; Galderisi et al., 2022) eliminating the need for biopsy‐derived samples. The aim of this study was to further validate C20/A4 cells as a cellular model for studying AKU in vitro and to develop, for the first time, an AKU inflammatory cell model. This model will be used to study the inflammatory processes associated with the disease, delving into the complex mechanisms involved in the formation of ochronotic pigment and its consequential impact on osteoarthropathy.

AKU, classified as a form of OA, implicates inflammatory conditions as pivotal in the clinical manifestation of the disease (Millucci et al., 2017). In our previous research, we demostrated that HGA promotes the production and release of SAA and pro‐inflammatory cytokines, along with oxidative stress, amyloid and ochronotic pigment formation, and cell death in an AKU cell model (Braconi et al., 2011; Braconi, Laschi, Taylor, et al., 2010; Tinti et al., 2010). However, to the best of our knowledge, this is the first report on the expression of inflammation biomarkers in C20/A4 human chondrocytes treated with HGA.

At first, our investigation revealed a notable increase in the production of NO, coupled with a heightened level of iNOS and COX‐2 enzymes. These findings highlighted the pro‐inflammatory environment induced by exposure to HGA.

Articular cartilage is a connective tissue that consists of a single type of cell named chondrocytes. These cells are surrounded by a self‐produced extracellular matrix (ECM), that contains water, collagen 2, agglomerated proteoglycans, and hydrophilic biological macromolecules. The ECM of cartilage has a dual function, serving both as a mechanical support and as a lubricant for bones and joints. The disruption of cartilage tissue structure in OA, and AKU, is associated with alterations in the molecular composition of ECM (Martel‐Pelletier et al., 2016). This degradation process is controlled by specific enzymes called aggrecanases, including A disintegrin and metalloproteinase with thrombospondin motifs−4 and −5, as well as collagenases such as matrix metalloproteases. The production of these enzymes is increased by NO (Wu et al., 2019). Additionally, a continuous increase in NO levels significantly inhibits the release of gelatinase and prostaglandins (PGs) in chondrocytes (Sun et al., 2018). Primarily, an excessive release of NO may compromise the structure of cartilage due to its regulatory influence on both proteoglycans and collagen metabolism. As reported by Jiang, H. et al. (Jiang et al., 2023), OA chondrocytes spontaneously secrete various inflammatory mediators such as IL‐1β, IL‐6, IL‐8, TNF‐α, and PGE2, whereas healthy chondrocytes do not release these mediators. It is widely recognized that iNOS plays a pivotal role in inflammatory processes. NO, a downstream signaling molecule of iNOS, acts as a potent reactive species at sites of inflammation. Furthermore, iNOS has been associated with the activity of COX‐2, as they both play a role in similar inflammatory pathways.

Several HGA‐related oxidative effects have been demonstrated in a wide range of AKU models in recent years. These includes the oxidation of chondrocytic proteins, evidenced by increased carbonylation and thiol oxidation (Braconi et al., 2012, 2015; Braconi, Laschi, Amato, et al., 2010; Braconi, Laschi, Taylor, et al., 2010; Tinti et al., 2010) and the production of a fluorescent melanin‐like ochronotic pigment in human serum, which could be partially counteracted by the use of antioxidants (Braconi et al., 2011; Braconi, Laschi, Amato, et al., 2010). Additionally, enhanced LPO (Braconi et al., 2011; Millucci et al., 2012; Millucci, Ghezzi, Paccagnini, et al., 2014; Spreafico et al., 2013a) and inflammation, as indicated by the release of pro‐inflammatory cytokines (Millucci, Ghezzi, Paccagnini, et al., 2014; Spreafico et al., 2013b) have also been observed. Although the oxidative stress biomarkers detected in AKU models are not specific to the disease, since these modifications are common in in many other disorders (Dalle‐Donne et al., 2006), their presence underscores the role of HGA as a potent pro‐oxidant compound.

Our study of cell inflammatory status during HGA‐induced ochronosis has revealed a marked increase in the generation of ROS, as demonstrated by heightened fluorescence intensity detected in H_2_DCFDA assay. This observation resonates with existing literature underscoring the pivotal role of ROS in governing chondrocyte homeostasis and extracellular matrix remodeling dynamics (Shen et al., 2021). NO can react with superoxide anions (O_2_^−^) to generate peroxynitrite, which is known to trigger inflammation and cell death in cartilage tissues. ROS could drive chondrocytes towards necrosis, while NO specifically induced apoptosis‐like morphological alterations (Blanco et al., 1995). Both NO and ROS are implicated in causing DNA damage, as evidenced by Chen's findings (Chen et al., 2008), revealing a significant increase in oxidative DNA damage in chondrocytes affected by OA. The progression of OA predominantly depends on ROS and oxidative stress. ROS are highly reactive molecules often considered harmful byproducts of cellular metabolism. However, recent research has revealed their complex and dual role in manteining and regulating cartilage homeostasis (Henrotin et al., 2003). Physiologically, ROS act as key signaling molecules which regulate chondrocyte function, ECM formation, and cartilage regeneration (Bardaweel et al., 2018). At low concentrations, ROS contribute to normal cellular activities such as proliferation, differentiation, and mechanical stress response, enabling cartilage to adaptively remodel itself. However, excessive ROS production, which is frequently induced by oxidative stress, might, lead to detrimental effects (Hong et al., 2024). Under oxidative stress, especially during inflammatory conditions, ROS production in cartilage can be significantly elevated. Inflammatory processes activate immune cells, including macrophages, which release pro‐inflammatory cytokines such as TNF‐α and IL‐1β. These cytokines, in turn, stimulate chondrocytes, leading to increased mitochondrial activity and ROS generation (Kan et al., 2021). ROS in cartilage are primarly produced as byproducts of the electron transport chain and through the activities of enzymes like NADPH oxidase (De Almeida et al., 2022). Elevated levels of ROS can cause oxidative damage to critical biological molecules, including DNA, lipids, and proteins, disrupting the delicate balance of cartilage homeostasis by damaging ECM components, such as collagen and proteoglycans (Bardaweel et al., 2018). This oxidative stress is typically associated with the development of degenerative joint conditions like OA, where ROS contribute to chondrocyte death, ECM degradation, and chronic inflammation.

Immunofluorescence and Western blot analysis demonstrate that 4‐HNE levels increased in C20/A4 human chondrocytes treated with HGA compared to control cells. It has been established that 4‐hydroxynonenal (4‐HNE), a by‐product of LPO, not only plays a crucial role in the degradation of cartilage in OA (Vaillancourt et al., 2008) but was also detected in both AKU cartilage and chondrocytes (Geminiani et al., 2017). These results further strengthen the evidence indicating the presence of oxidative stress in cells treated with HGA.

Due to the established connection between AKU and amyloidosis (Millucci et al., 2015), the well‐known role of HGA in the development of AKU‐associated SAA amyloidosis (Braconi et al., 2017), as well as the common production of SAA during inflammatory responses (Sorić Hosman et al., 2021), we have choosen to investigate its expression in C20/A4 human chondrocytes upon stimulation with HGA. Our results revealed that there was an increase of SAA levels in both the control and treated cells. The two proteins were evenly distributed in control C20/A4 human chondrocytes; prior studies have addressed the intrinsic production of SAA of these cells as a consequence of their tumorigenic characteristics (Lee & Beatty, 2021; Lin et al., 2019; Malle et al., 2009; Moshkovskii, 2012). Nevertheless, when the cells are exposed to HGA, SAA and SAP become mostly concentrated in certain areas, particularly around the nucleus, confirming the role of HGA in the aggregation of SAA, but also indicating a possible physical connection between SAA and SAP. Similarly, we noted a comparable pattern for SAP. These findings were further validated by Congo Red staining, which revealed the presence of amyloid fibril structures exclusively in HGA‐treated cells, as indicated by green/yellow staining, while such structures were absent in control cells. Our hypothesis suggests that although SAA and SAP proteins are synthesized in control cells, their interaction, ultimately leading to amyloid formation, does not occur in the absence of HGA‐induced effects. This is supported by the lack of staining observed under polarized light by Congo Red staining, indicative of the inability to facilitate aggregation and subsequent amyloid fibril formation in the absence of HGA.

In conclusion, this study reveals a novel link between inflammation and AKU progression, providing new insights into the disease's underlying mechanisms. Our findings have significantly advanced our understanding of the pathophysiological processes, particularly HGA‐induced ochronosis and its association with inflammatory pathways, using a novel cellular model. This model offers a more efficient and valuable approach for examining disease‐related events and developing targeted therapeutic options. Future research should build on these findings to further investigate AKU progression and explore targeted anti‐inflammatory therapies that could alleviate or prevent ochronosis‐related pain in AKU patients.

## AUTHOR CONTRIBUTIONS

Pierfrancesco Mastroeni, Michela Geminiani conceived and designed the experiments. Pierfrancesco Mastroeni, Tommaso Olmastroni and Luisa Frusciante conducted the experiments. Pierfrancesco Mastroeni and Michela Geminiani analyzed the data. Pierfrancesco Mastroeni, Michela Geminiani and Annalisa Santucci wrote the manuscript. Annalisa Santucci supervised on the study. Alfonso Trezza and Anna Visibelli visualized and revisioned the manuscript. All authors reviewed and approved the final manuscript.

## CONFLICT OF INTEREST STATEMENT

The authors declare no competing interests.

## References

[jcp31449-bib-0001] De Almeida, A. J. P. O. , De Oliveira, J. C. P. L. , Da Silva Pontes, L. V. , De Souza Júnior, J. F. , Gonçalves, T. A. F. , Dantas, S. H. , De Almeida Feitosa, M. S. , Silva, A. O. , & De Medeiros, I. A. (2022). ROS: Basic concepts, sources, cellular signaling, and its implications in aging pathways, Oxidative medicine and cellular longevity (2022). Hindawi Limited. 10.1155/2022/1225578 PMC9605829 36312897

[jcp31449-bib-0002] Bardaweel, S. K. , Gul, M. , Alzweiri, M. , Ishaqat, A. , Alsalamat, H. A. , & Bashatwah, R. M. (2018). Reactive oxygen species: The dual role in physiological and pathological conditions of the human body. Eurasian Journal of Medicine, 50, 193–201. 10.5152/eurasianjmed.2018.17397 30515042 PMC6263229

[jcp31449-bib-0003] Bernardini, G. , Braconi, D. , Spreafico, A. , & Santucci, A. (2012). Post‐genomics of bone metabolic dysfunctions and neoplasias. Proteomics, 12(Issues 4–5), 708–721. 10.1002/pmic.201100358 22246652

[jcp31449-bib-0004] Bernardini, G. , Braconi, D. , Zatkova, A. , Sireau, N. , Kujawa, M. J. , Introne, W. J. , Spiga, O. , Geminiani, M. , Gallagher, J. A. , Ranganath, L. R. , & Santucci, A. (2024). Alkaptonuria. Nature Reviews Disease Primers, 10(1), 16. 10.1038/s41572-024-00498-x 38453957

[jcp31449-bib-0005] Blanco, F. J. , Ochs, R. L. , Schwarz, H. , & Lotz, M. (1995). Chondrocyte apoptosis induced by nitric oxide. The American Journal of Pathology, 146(1), 75–85. 7856740 PMC1870754

[jcp31449-bib-0006] Braconi, D. , Bernardini, G. , Bianchini, C. , Laschi, M. , Millucci, L. , Amato, L. , Tinti, L. , Serchi, T. , Chellini, F. , Spreafico, A. , & Santucci, A. (2012). Biochemical and proteomic characterization of alkaptonuric chondrocytes. Journal of Cellular Physiology, 227(9), 3333–3343. 10.1002/jcp.24033 22213341 PMC3427902

[jcp31449-bib-0007] Braconi, D. , Bernardini, G. , Paffetti, A. , Millucci, L. , Geminiani, M. , Laschi, M. , Frediani, B. , Marzocchi, B. , & Santucci, A. (2016). Comparative proteomics in alkaptonuria provides insights into inflammation and oxidative stress. The International Journal of Biochemistry & Cell Biology, 81, 271–280. 10.1016/J.BIOCEL.2016.08.016 27590860

[jcp31449-bib-0008] Braconi, D. , Bianchini, C. , Bernardini, G. , Laschi, M. , Millucci, L. , Spreafico, A. , & Santucci, A. (2011). Redox‐proteomics of the effects of homogentisic acid in an in vitro human serum model of alkaptonuric ochronosis. Journal of Inherited Metabolic Disease, 34(6), 1163–1176. 10.1007/s10545-011-9377-6 21874298

[jcp31449-bib-0009] Braconi, D. , Laschi, M. , Amato, L. , Bernardini, G. , Millucci, L. , Marcolongo, R. , Cavallo, G. , Spreafico, A. , & Santucci, A. (2010). Evaluation of anti‐oxidant treatments in an in vitro model of alkaptonuric ochronosis. Rheumatology, 49(10), 1975–1983. 10.1093/rheumatology/keq.175 20601653

[jcp31449-bib-0010] Braconi, D. , Laschi, M. , Taylor, A. M. , Bernardini, G. , Spreafico, A. , Tinti, L. , Gallagher, J. A. , & Santucci, A. (2010). Proteomic and redox‐proteomic evaluation of homogentisic acid and ascorbic acid effects on human articular chondrocytes. Journal of Cellular Biochemistry, 111(4), 922–932. 10.1002/jcb.22780 20665660

[jcp31449-bib-0011] Braconi, D. , Millucci, L. , Bernardini, G. , & Santucci, A. (2015). Oxidative stress and mechanisms of ochronosis in alkaptonuria, Free Radical Biology and Medicine (88, pp. 70–80). Elsevier Inc. 10.1016/j.freeradbiomed.2015.02.021 25733348

[jcp31449-bib-0012] Braconi, D. , Millucci, L. , Bernini, A. , Spiga, O. , Lupetti, P. , Marzocchi, B. , Niccolai, N. , Bernardini, G. , & Santucci, A. (2017). Homogentisic acid induces aggregation and fibrillation of amyloidogenic proteins. Biochimica et Biophysica Acta (BBA) ‐ General Subjects, 1861(2), 135–146. 10.1016/j.bbagen.2016.11.026 27865997

[jcp31449-bib-0013] Chen, A. F. , Davies, C. M. , De Lin, M. , & Fermor, B. (2008). Oxidative DNA damage in osteoarthritic porcine articular cartilage. Journal of Cellular Physiology, 217(3), 828–833. 10.1002/jcp.21562 18720406 PMC2575799

[jcp31449-bib-0014] Dalle‐Donne, I. , Rossi, R. , Colombo, R. , Giustarini, D. , & Milzani, A. (2006). Biomarkers of oxidative damage in human disease. Clinical Chemistry, 52(Issue 4), 601–623. 10.1373/clinchem.2005.061408 16484333

[jcp31449-bib-0015] Feoktistova, M. , Geserick, P. , & Leverkus, M. (2016). Crystal violet assay for determining viability of cultured cells. Cold Spring Harbor Protocols, 2016(4), pdb.prot087379. 10.1101/pdb.prot087379 27037069

[jcp31449-bib-0016] Fisher, A. A. , & Davis, M. W. (2004). Alkaptonuric ochronosis with aortic valve and joint replacements and femoral fracture: A case report and literature review. Clinical Medicine & Research, 2(4), 209–215. 10.3121/cmr.2.4.209 15931360 PMC1069096

[jcp31449-bib-0017] Forrester, S. J. , Kikuchi, D. S. , Hernandes, M. S. , Xu, Q. , & Griendling, K. K. (2018). Reactive oxygen species in metabolic and inflammatory signaling. Circulation Research, 122(6), 877–902. 10.1161/CIRCRESAHA.117.311401 29700084 PMC5926825

[jcp31449-bib-0018] Galderisi, S. , Milella, M. S. , Rossi, M. , Cicaloni, V. , Rossi, R. , Giustarini, D. , Spiga, O. , Tinti, L. , Salvini, L. , Tinti, C. , Braconi, D. , Millucci, L. , Lupetti, P. , Prischi, F. , Bernardini, G. , & Santucci, A. (2022). Homogentisic acid induces autophagy alterations leading to chondroptosis in human chondrocytes: Implications in alkaptonuria. Archives of Biochemistry and Biophysics, 717, 109137. 10.1016/j.abb.2022.109137 35090868

[jcp31449-bib-0019] Gallagher, J. A. , Ranganath, L. R. , & Boyde, A. (2015). Lessons from rare diseases of cartilage and bone. Current Opinion in Pharmacology, 22, 107–114. 10.1016/j.coph.2015.04.002 25978274

[jcp31449-bib-0020] Gambassi, S. , Geminiani, M. , Thorpe, S. D. , Bernardini, G. , Millucci, L. , Braconi, D. , Orlandini, M. , Thompson, C. L. , Petricci, E. , Manetti, F. , Taddei, M. , Knight, M. M. , & Santucci, A. (2017). Smoothened‐antagonists reverse homogentisic acid‐induced alterations of Hedgehog signaling and primary cilium length in alkaptonuria. Journal of Cellular Physiology, 232(11), 3103–3111. 10.1002/JCP.25761 28019670

[jcp31449-bib-0021] Geminiani, M. , Gambassi, S. , Millucci, L. , Lupetti, P. , Collodel, G. , Mazzi, L. , Frediani, B. , Braconi, D. , Marzocchi, B. , Laschi, M. , Bernardini, G. , & Santucci, A. (2017). Cytoskeleton aberrations in alkaptonuric chondrocytes. Journal of Cellular Physiology, 232, 1728–1738. 10.1002/jcp.25500 27454006

[jcp31449-bib-0023] Hara, H. , Naito, M. , Harada, T. , Tsuboi, I. , Terui, T. , & Aizawa, S. (2016). Quantitative analysis of formaldehyde‐induced fluorescence in paraffin‐embedded specimens of malignant melanomas and other melanocytic lesions. Acta Dermato Venereologica, 96(3), 309–313. 10.2340/00015555-2238 26350793

[jcp31449-bib-0024] Helliwell, T. R. , Gallagher, J. A. , & Ranganath, L. (2008). Alkaptonuria ‐ A review of surgical and autopsy pathology. Histopathology, 53(5), 503–512. 10.1111/j.1365-2559.2008.03000.x 18336562

[jcp31449-bib-0025] Henrotin, Y. E. , Bruckner, P. , & Pujol, J. P. L. (2003). The role of reactive oxygen species in homeostasis and degradation of cartilage. Osteoarthritis and Cartilage, 11(10), 747–755. 10.1016/S1063-4584(03)00150-X 13129694

[jcp31449-bib-0026] Hong, Y. , Boiti, A. , Vallone, D. , & Foulkes, N. S. (2024). Reactive oxygen species signaling and oxidative stress: Transcriptional regulation and evolution. Antioxidants, 13(3), 312. 10.3390/antiox13030312 38539845 PMC10967436

[jcp31449-bib-0027] Jiang, H. , Ji, P. , Shang, X. , & Zhou, Y. (2023). Connection between osteoarthritis and nitric oxide: From pathophysiology to therapeutic target. Molecules, 28(4):1683. 10.3390/molecules28041683 36838671 PMC9959782

[jcp31449-bib-0028] Kan, S. , Duan, M. , Liu, Y. , Wang, C. , & Xie, J. (2021). Role of mitochondria in physiology of chondrocytes and diseases of osteoarthritis and rheumatoid arthritis. Cartilage, 13(2), 1102S–1121S. 10.1177/19476035211063858 34894777 PMC8804744

[jcp31449-bib-0029] Laschi, M. , Tinti, L. , Braconi, D. , Millucci, L. , Ghezzi, L. , Amato, L. , Selvi, E. , Spreafico, A. , Bernardini, G. , & Santucci, A. (2012). Homogentisate 1,2 dioxygenase is expressed in human osteoarticular cells: Implications in alkaptonuria. Journal of Cellular Physiology, 227(9), 3254–3257. 10.1002/jcp.24018 22105303 PMC3427883

[jcp31449-bib-0030] Lee, J. , & Beatty, G. L. (2021). Serum amyloid A proteins and their impact on metastasis and immune biology in cancer. Cancers, 13(13), 3179. 10.3390/cancers13133179 34202272 PMC8267706

[jcp31449-bib-0031] Lin, H. , Tan, G. , Liu, Y. , & Lin, S. (2019). The prognostic value of serum amyloid A in solid tumors: A meta‐analysis. Cancer Cell International, 19(1), 62. 10.1186/s12935-019-0783-4 30930691 PMC6425599

[jcp31449-bib-0032] Malle, E. , Sodin‐Semrl, S. , & Wcislo‐Dziadecka, A. (2009). Serum amyloid A: An acute‐phase protein involved in tumour pathogenesis, Cellular and molecular life sciences (Vol. 66 pp. 9–26). Birkhauser Verlag Basel. 10.1007/s00018-008-8321-x 18726069 PMC4864400

[jcp31449-bib-0033] Martel‐Pelletier, J. , Barr, A. J. , Cicuttini, F. M. , Conaghan, P. G. , Cooper, C. , Goldring, M. B. , Goldring, S. R. , Jones, G. , Teichtahl, A. J. , & Pelletier, J. P. (2016). Osteoarthritis, Nature reviews disease primers (2). Nature Publishing Group. 10.1038/nrdp.2016.72 27734845

[jcp31449-bib-0034] Millucci, L. , Bernardini, G. , Spreafico, A. , Orlandini, M. , Braconi, D. , Laschi, M. , Geminiani, M. , Lupetti, P. , Giorgetti, G. , Viti, C. , Frediani, B. , Marzocchi, B. , & Santucci, A. (2017). Histological and ultrastructural characterization of alkaptonuric tissues. Calcified Tissue International, 101(1), 50–64. 10.1007/S00223-017-0260-9/FIGURES/8 28271171

[jcp31449-bib-0035] Millucci, L. , Braconi, D. , Bernardini, G. , Lupetti, P. , Rovensky, J. , Ranganath, L. , & Santucci, A. (2015). Amyloidosis in alkaptonuria. Journal of Inherited Metabolic Disease, 38(5), 797–805. 10.1007/S10545-015-9842-8 25868666

[jcp31449-bib-0036] Millucci, L. , Ghezzi, L. , Bernardini, G. , Braconi, D. , Lupetti, P. , Perfetto, F. , Orlandini, M. , & Santucci, A. (2014). Diagnosis of secondary amyloidosis in alkaptonuria. Diagnostic Pathology, 9(1), 185. 10.1186/s13000-014-0185-9 25567001 PMC4189149

[jcp31449-bib-0037] Millucci, L. , Ghezzi, L. , Braconi, D. , Laschi, M. , Geminiani, M. , Amato, L. , Orlandini, M. , Benvenuti, C. , Bernardini, G. , & Santucci, A. (2014). Secondary amyloidosis in an alkaptonuric aortic valve. International Journal of Cardiology, 172(1), e121–e123. 10.1016/J.IJCARD.2013.12.117 24456888 PMC3991337

[jcp31449-bib-0038] Millucci, L. , Ghezzi, L. , Paccagnini, E. , Giorgetti, G. , Viti, C. , Braconi, D. , Laschi, M. , Geminiani, M. , Soldani, P. , Lupetti, P. , Orlandini, M. , Benvenuti, C. , Perfetto, F. , Spreafico, A. , Bernardini, G. , & Santucci, A. (2014). Amyloidosis, inflammation, and oxidative stress in the heart of an alkaptonuric patient. Mediators of Inflammation, 2014, 1–12. 10.1155/2014/258471 PMC4020161 24876668

[jcp31449-bib-0039] Millucci, L. , Giorgetti, G. , Viti, C. , Ghezzi, L. , Gambassi, S. , Braconi, D. , Marzocchi, B. , Paffetti, A. , Lupetti, P. , Bernardini, G. , Orlandini, M. , & Santucci, A. (2015). Chondroptosis in alkaptonuric cartilage. Journal of Cellular Physiology, 230(5), 1148–1157. 10.1002/jcp.24850 25336110 PMC5024069

[jcp31449-bib-0040] Millucci, L. , Spreafico, A. , Tinti, L. , Braconi, D. , Ghezzi, L. , Paccagnini, E. , Bernardini, G. , Amato, L. , Laschi, M. , Selvi, E. , Galeazzi, M. , Mannoni, A. , Benucci, M. , Lupetti, P. , Chellini, F. , Orlandini, M. , & Santucci, A. (2012). Alkaptonuria is a novel human secondary amyloidogenic disease. Biochimica et Biophysica Acta (BBA) ‐ Molecular Basis of Disease, 1822(11), 1682–1691. 10.1016/j.bbadis.2012.07.011 22850426 PMC3787765

[jcp31449-bib-0041] Momohara, S. , Okamoto, H. , & Yamanaka, H. (2008). Chondrocyte of rheumatoid arthritis serve as a source of intra‐articular acute‐phase serum amyloid A protein. Clinica Chimica Acta, 398, 155–156. 10.1016/j.cca.2008.07.034 18755174

[jcp31449-bib-0042] Moshkovskii, S. A. (2012). Why do cancer cells produce serum amyloid a acute‐phase protein? Biochemistry (Moscow) (77, pp. 339–341). Maik Nauka Publishing/Springer SBM. Issue 4. 10.1134/S0006297912040037 22809151

[jcp31449-bib-0043] Ng, N. , & Ooi, L. (2021). A simple microplate assay for reactive oxygen species generation and rapid cellular protein normalization. Bio‐Protocol, 11(1), e3877. 10.21769/BioProtoc.3877 33732765 PMC7952951

[jcp31449-bib-0022] Phornphutkul, C. , Introne, W. J. , Perry, M. B. , Bernardini, I. , Murphey, M. D. , Fitzpatrick, D. L. , Anderson, P. D. , Huizing, M. , Anikster, Y. , Gerber, L. H. , & Gahl, W. A. (2002). Natural history of alkaptonuria. The New England Journal of Medicine, 347(26), 2111–2121. 10.1056/NEJMoa021736 12501223

[jcp31449-bib-0044] Ranganath, L. R. , Milan, A. M. , Hughes, A. T. , Dutton, J. J. , Fitzgerald, R. , Briggs, M. C. , Bygott, H. , Psarelli, E. E. , Cox, T. F. , Gallagher, J. A. , Jarvis, J. C. , van Kan, C. , Hall, A. K. , Laan, D. , Olsson, B. , Szamosi, J. , Rudebeck, M. , Kullenberg, T. , Cronlund, A. , … Rovensky, J. (2016). Suitability of nitisinone in alkaptonuria 1 (SONIA 1): An international, multicentre, randomised, open‐label, no‐treatment controlled, parallel‐group, dose‐response study to investigate the effect of once daily nitisinone on 24‐h urinary homogentisic acid excretion in patients with alkaptonuria after 4 weeks of treatment. Annals of the Rheumatic Diseases, 75(2), 362–367. 10.1136/annrheumdis-2014-206033 25475116

[jcp31449-bib-0045] Ranganath, L. R. , Psarelli, E. E. , Arnoux, J.‐B. , Braconi, D. , Briggs, M. , Bröijersén, A. , Loftus, N. , Bygott, H. , Cox, T. F. , Davison, A. S. , Dillon, J. P. , Fisher, M. , FitzGerald, R. , Genovese, F. , Glasova, H. , Hall, A. K. , Hughes, A. T. , Hughes, J. H. , Imrich, R. , … Gallagher, J. A. (2020). Efficacy and safety of once‐daily nitisinone for patients with alkaptonuria (SONIA 2): An international, multicentre, open‐label, randomised controlled trial. The Lancet Diabetes & Endocrinology, 8, 762–772. 10.1016/S2213-8587(20)30228-X 32822600

[jcp31449-bib-0046] Shen, P. C. , Chou, S. H. , Lu, C. C. , Huang, H. T. , Chien, S. H. , Huang, P. J. , Liu, Z. M. , Shih, C. L. , Su, S. J. , Chen, L. M. , & Tien, Y. C. (2021). Shockwave treatment enhanced extracellular matrix production in articular chondrocytes through activation of the ROS/MAPK/Nrf2 signaling pathway. Cartilage, 13(2), 238S–253S. 10.1177/19476035211012465 34238028 PMC8804851

[jcp31449-bib-0047] Sorić Hosman, I. , Kos, I. , & Lamot, L. (2021). Serum amyloid A in Inflammatory rheumatic diseases: A compendious review of a renowned biomarker. Frontiers in Immunology, 11, 631299. 10.3389/fimmu.2020.631299 33679725 PMC7933664

[jcp31449-bib-0048] Spreafico, A. , Millucci, L. , Ghezzi, L. , Geminiani, M. , Braconi, D. , Amato, L. , Chellini, F. , Frediani, B. , Moretti, E. , Collodel, G. , Bernardini, G. , & Santucci, A. (2013a). Antioxidants inhibit SAA formation and pro‐inflammatory cytokine release in a human cell model of alkaptonuria. Rheumatology (Oxford, England), 52(9), 1667. 10.1093/RHEUMATOLOGY/KET185 23704321 PMC3741479

[jcp31449-bib-0049] Spreafico, A. , Millucci, L. , Ghezzi, L. , Geminiani, M. , Braconi, D. , Amato, L. , Chellini, F. , Frediani, B. , Moretti, E. , Collodel, G. , Bernardini, G. , & Santucci, A. (2013b). Antioxidants inhibit SAA formation and pro‐inflammatory cytokine release in a human cell model of alkaptonuria. Rheumatology (United Kingdom), 52(9), 1667–1673. 10.1093/rheumatology/ket185 PMC3741479 23704321

[jcp31449-bib-0050] Sun, E. Y. , Fleck, A. K. M. , Abu‐Hakmeh, A. E. , Kotsakis, A. , Leonard, G. R. , & Wan, L. Q. (2018). Cartilage metabolism is modulated by synovial fluid through metalloproteinase activity. Annals of Biomedical Engineering, 46(6), 810–818. 10.1007/s10439-018-2010-1 29589167

[jcp31449-bib-0051] Thorpe, S. D. , Gambassi, S. , Thompson, C. L. , Chandrakumar, C. , Santucci, A. , & Knight, M. M. (2017). Reduced primary cilia length and altered Arl13b expression are associated with deregulated chondrocyte Hedgehog signaling in alkaptonuria. Journal of Cellular Physiology, 232(9), 2407–2417. 10.1002/jcp.25839 28158906 PMC5484994

[jcp31449-bib-0052] Tinti, L. , Spreafico, A. , Braconi, D. , Millucci, L. , Bernardini, G. , Chellini, F. , Cavallo, G. , Selvi, E. , Galeazzi, M. , Marcolongo, R. , Gallagher, J. A. , & Santucci, A. (2010). Evaluation of antioxiodant drugs for the treatment of ochronotic alkaptonuria in an in vitro human cell model. Journal of Cellular Physiology, 225(1), 84–91. 10.1002/JCP.22199 20648626

[jcp31449-bib-0053] Tinti, L. , Taylor, A. M. , Santucci, A. , Wlodarski, B. , Wilson, P. J. , Jarvis, J. C. , Fraser, W. D. , Davidson, J. S. , Ranganath, L. R. , & Gallagher, J. A. (2011). Development of an in vitro model to investigate joint ochronosis in alkaptonuria. Rheumatology, 50(2), 271–277. 10.1093/rheumatology/keq.246 20952450

[jcp31449-bib-0054] Vaillancourt, F. , Fahmi, H. , Shi, Q. , Lavigne, P. , Ranger, P. , Fernandes, J. C. , & Benderdour, M. (2008). 4‐Hydroxynonenal induces apoptosis in human osteoarthritic chondrocytes: The protective role of glutathione‐S‐transferase. Arthritis Research & Therapy, 10(5), R107. 10.1186/ar2503 18782442 PMC2592788

[jcp31449-bib-0055] Wu, Y. , Lin, Z. , Yan, Z. , Wang, Z. , Fu, X. , & Yu, K. (2019). Sinomenine contributes to the inhibition of the inflammatory response and the improvement of osteoarthritis in mouse‐cartilage cells by acting on the Nrf2/HO‐1 and NF‐κB signaling pathways. International Immunopharmacology, 75, 105715. 10.1016/j.intimp.2019.105715 31310911

